# Sickness Absence from Work among Persons with New Physician-Diagnosed Carpal Tunnel Syndrome: A Population-Based Matched-Cohort Study

**DOI:** 10.1371/journal.pone.0119795

**Published:** 2015-03-24

**Authors:** Isam Atroshi, Caddie Zhou, Anna Jöud, Ingemar F. Petersson, Martin Englund

**Affiliations:** 1 Orthopedics, Department of Clinical Sciences Lund, Lund University, Lund, Sweden; 2 Department of Orthopedics Hässleholm-Kristianstad, Hässleholm Hospital, Hässleholm, Sweden; 3 Epidemiology and Register Centre South, Skåne University Hospital Lund, Lund, Sweden; 4 Division of Occupational and Environmental Medicine, Department of Laboratory Medicine, Lund University, Lund, Sweden; 5 Clinical Epidemiology Research & Training Unit, Boston University School of Medicine, Boston, Massachusetts, United States of America; institute of Health Policy and Management, NETHERLANDS

## Abstract

**Background:**

Carpal tunnel syndrome is common among employed persons. Data on sickness absence from work in relation to carpal tunnel syndrome have been usually based on self-report and derived from clinical or occupational populations. We aimed to determine sickness absence among persons with physician-diagnosed carpal tunnel syndrome as compared to the general population.

**Methods:**

In Skåne region in Sweden we identified all subjects, aged 17–57 years, with new physician-made diagnosis of carpal tunnel syndrome during 5 years (2004–2008). For each subject we randomly sampled, from the general population, 4 matched reference subjects without carpal tunnel syndrome; the two cohorts comprised 5456 and 21,667 subjects, respectively (73% women; mean age 43 years). We retrieved social insurance register data on all sickness absence periods longer than 2 weeks from 12 months before to 24 months after diagnosis. Of those with carpal tunnel syndrome 2111 women (53%) and 710 men (48%) underwent surgery within 24 months of diagnosis. We compared all-cause sickness absence and analyzed sickness absence in conjunction with diagnosis and surgery.

**Results:**

Mean number of all-cause sickness absence days per each 30-day period from 12 months before to 24 months after diagnosis was significantly higher in the carpal tunnel syndrome than in the reference cohort. A new sickness absence period longer than 2 weeks in conjunction with diagnosis was recorded in 12% of the women (n = 492) and 11% of the men (n = 170) and with surgery in 53% (n = 1121) and 58% (n = 408) of the surgically treated, respectively; median duration in conjunction with surgery was 35 days (IQR 27–45) for women and 41 days (IQR 28–50) for men.

**Conclusions:**

Persons with physician-diagnosed carpal tunnel syndrome have substantially more sickness absence from work than age and sex-matched persons from the general population from1 year before to 2 years after diagnosis. Gender differences were small.

## Introduction

Carpal tunnel syndrome (CTS) is a common cause of hand symptoms and activity limitations among people of working age [1–3]. More than a third of patients with CTS undergo surgical treatment [3,4]. After surgery, the mean duration of sickness absence from work, usually related to the postoperative morbidity, has been approximately 4 weeks [5]. However, a proportion of patients may have substantially more prolonged work incapacity. In a study of more than 127,000 patients who had surgery for CTS in France in 2008, 37% had still not returned to work by 8 weeks after surgery [6]. To our knowledge no data about sickness absence from work related to nonsurgically treated CTS have been published.

Data on CTS-related sickness absence from work has been reported in observational studies that usually involved specific clinical or occupational populations and in a few randomized clinical trials [7]. In almost all studies sickness absence data were based on patient self-report. The agreement between self-report data and public register data regarding duration of sickness absence from work has been found to be poor [8]. The incidence and duration of CTS-related sickness absence from work, including potential gender aspects, have not been addressed in population-based studies. Such information would be helpful in planning measures aimed at reducing CTS-related work disability.

We conducted a population-based study of a cohort with new physician-diagnosed CTS and a matched reference cohort without CTS to determine CTS-related sickness absence from work as compared to that in the general population.

## Materials and Methods

### Study Cohorts

We derived the study cohorts from the population of the Skåne region in southern Sweden (1.2 million inhabitants, one-eighth of the population of Sweden). All inpatient and outpatient healthcare provided in the region is registered in the Skåne Healthcare Register (SHR). After each patient visit to a doctor, the diagnosis is registered by the doctor according to the International Classification of Diseases and Related Health Problems 10 (ICD-10) system. From the SHR we retrieved data on all persons who had received a physician-made diagnosis of CTS during a 5-year period (January 1, 2004 through December 31, 2008). The inclusion criteria were (1) age at diagnosis from 17 years (16 years is the minimum age eligible for sick-leave payment) to 57 years (chosen because of the relatively high rate of early retirement or disability pension among individuals over 60 years), (2) a primary diagnosis of CTS made by a medical doctor, and (3) resident in the region during 3 calendar years before the date of diagnosis. We excluded persons who had received a diagnosis of CTS during the 3 years before the date of first CTS diagnosis registered during the study period.

For each subject with CTS we randomly sampled 4 matched reference subjects from the general population. The matching variables were sex, year of birth and district of residence. The date of diagnosis for each CTS subject was considered as day 0 for her/his matched reference subjects. The reference subjects had to be residents in the region and with no CTS diagnosis.

### Sickness Absence from Work

In Sweden, every individual (legally working or is registered as unemployed) who cannot work owing to illness or injury is entitled to sickness benefit starting on day 2 of the reported sickness period. For working individuals, sickness benefit from day 2 to day 14 is paid for by the employer. The Swedish social insurance, covering all residents, is administered by the Swedish Social Insurance Agency (SSIA). All data on sickness absence periods lasting longer than 14 days are registered by the SSIA in the Social Insurance Register. All sickness absence from work exceeding 7 days must be granted by a doctor. For employed individuals with a sickness absence exceeding 14 days, data for that period are registered from day 2. For employed individuals with a sickness absence lasting 1 to 14 days, that period is not registered by the SSIA. We retrieved data on sickness absence from work for the CTS and reference cohorts from the social insurance register. For each individual, we retrieved sickness absence data from 12 months before to 24 months after the date of diagnosis for the CTS subject and the corresponding date for the reference subjects.

### Analysis

Because absence from work due to sickness can be granted for either a full working day (ie 8 hours) or for three-quarters, half, or one-quarter, we calculated the net full-time sickness absence days. We grouped the CTS subjects according to treatment; individuals who underwent carpal tunnel release (received procedure code ACC51 within 24 months after date of first diagnosis) were included in the CTS surgical subcohort and the remaining individuals in the nonsurgical subcohort. We then analyzed sickness absence from work according to treatment. All analyses were sex-specific.

First, we analyzed all sickness absence periods irrespective of underlying cause (all-cause sickness absence). We calculated the mean number of days of sickness absence from work (and 95% CI) per each 30-day period from 12 months before to 24 months after the date of first diagnosis for the CTS cohort and corresponding date for the reference cohort. Second, we analyzed new sickness absence periods that started from 15 days before to 7 days after a visit to a physician in which CTS was registered as the primary diagnosis or date of carpal tunnel release. We calculated the mean and standard deviation (SD) and median and inter-quartile range (IQR) days of sickness absence in conjunction with the date of first diagnosis and the date of surgery. Finally, we calculated the total number of days of sickness absence in conjunction with any doctor visit in which a CTS diagnosis was given, up to 24 months after the date of first diagnosis.

### Ethics Statement

The study was conducted according to the Declaration of Helsinki and was approved by the Ethical Review Board of Lund University, Sweden. The population in the Skåne region was informed of the study and offered “opt-out” via regional news press, a process sanctioned by the Ethical Review Board.

## Results

During the 5 calendar years a physician-made primary diagnosis of CTS was given to 7108 residents aged 17 to 57 years. We excluded 1641 persons because they had previously received a CTS diagnosis during the 3 years preceding the date of first diagnosis in the study period. We excluded 11 persons because no eligible matched reference subjects could be found. Hence, the final cohort comprised 5456 subjects with CTS (73% women) and 21,667 matched reference subjects without CTS diagnosis. The mean age for the CTS cohort and the reference cohort was 43 years (range 17–57 years) for both sexes and approximately half the cohorts belonged to the age group 45 to 57 years (Table 1). Of the CTS cohort, 2111 women (53%) and 710 men (48%) underwent surgical treatment within 24 months of diagnosis (ACC51 was the primary code in 95%).

**Table 1:** Characteristics of the carpal tunnel syndrome (CTS) cohort and the reference cohort.

Age group	CTS cohort		Reference cohort	
(yrs)	Women	Men	Women	Men
**17–24**	147 (3.7)	47 (3.2)	584 (3.7)	186 (3.1)
**25–34**	710 (17.9)	261 (17.5)	2797 (17.8)	1029 (17.4)
**35–44**	1250 (31.5)	436 (29.2)	4978 (31.6)	1728 (29.2)
**45–57**	1859 (46.9)	746 (50.1)	7397 (46.9)	2968 (50.2)
***All***	*3966 (72*.*7)*	*1490 (27*.*3)*	*15756 (72*.*7)*	*5911 (27*.*3)*

All values are n (%).

### All-Cause Sickness Absence

**Fig 1:**
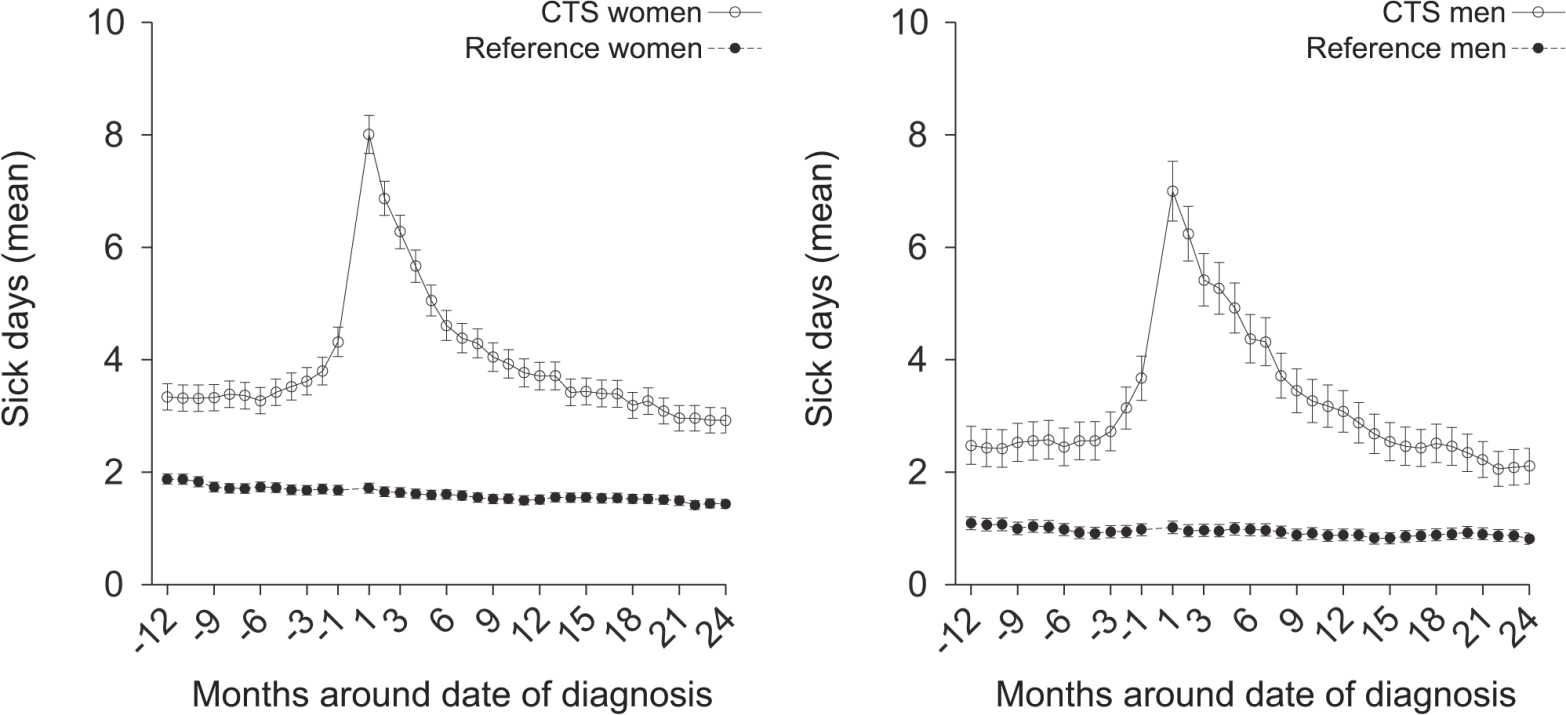
All-cause sickness absence from work around date of first diagnosis. Graphs showing the mean net (full-time) days of all-cause sickness absence per each 30-day period from 12 months before to 24 months after date of first diagnosis for the carpal tunnel syndrome (CTS) cohort and the reference cohort; the date of diagnosis for each CTS subject considered as day 0 for her/his matched reference subjects.

In both women and men the mean number of days of all-cause sickness absence from work per each 30-day period from 12 months before to 24 months after the date of first diagnosis was significantly higher in the CTS cohort than in the reference cohort (Fig. 1). The mean number of days of sickness absence per each 30-day period was initially about 1.5 days higher in the CTS cohort. The difference increased sharply at about 1 month before the date of diagnosis to a peak of approximately 6.5 days and then decreased during the following months. The difference between the groups at 24 months after diagnosis was similar to the initial difference. In both the CTS and reference cohorts the mean number of days of all-cause sickness absence per each 30-day period from 12 months before to 24 months after diagnosis was about 0.5 to 1 day higher in women than in men throughout the 36 months.

**Fig 2:**
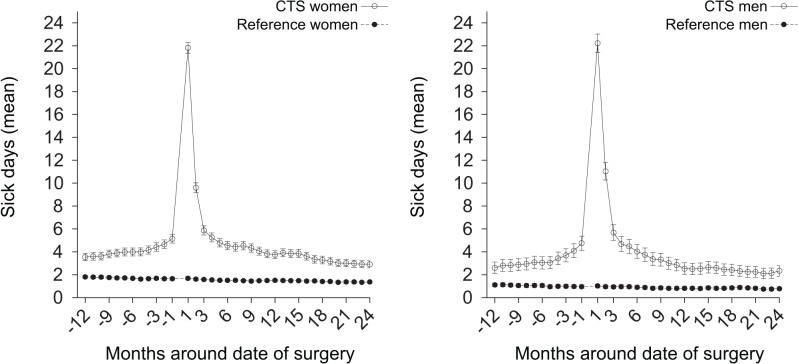
All-cause sickness absence from work around date of surgery. Graphs showing the mean net (full-time) days of all-cause sickness absence per each 30-day period from 12 months before to 24 months after date of surgery for the carpal tunnel syndrome (CTS) surgical subcohort and the reference cohort; the date of surgery for each CTS subject considered as day 0 for her/his matched reference subjects.

In the CTS surgical subcohort sickness absence started to increase sharply 1 month before the date of surgery, peaking at 2 months after surgery and then decreased sharply at 3 months (Fig. 2).

**Fig 3:**
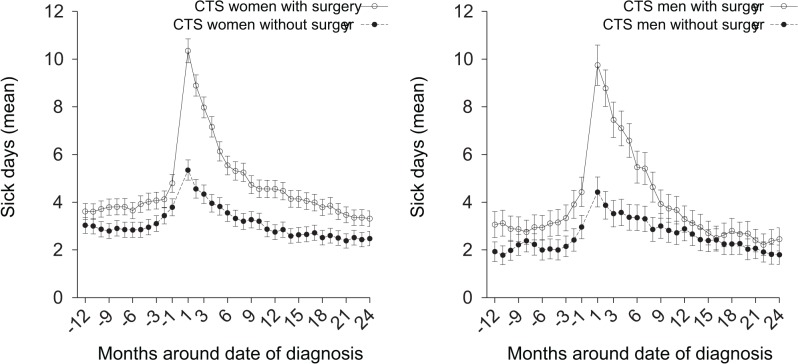
All-cause sickness absence from work around date of first diagnosis according to surgery. Graphs showing the mean net (full-time) days of all-cause sickness absence per each 30-day period from 12 months before to 24 months after date of first diagnosis for the carpal tunnel syndrome (CTS) surgical and nonsurgical subcohorts.

The mean number of days of sickness absence per each 30-day period was consistently higher in the surgical subcohort than in the nonsurgical subcohort; the difference was largest at 1 to 2 month after the date of diagnosis (Fig. 3).

### Sickness Absence in Conjunction with New Diagnosis of Carpal Tunnel Syndrome and Surgery

In the whole CTS cohort (3966 women and 1490 men), a new period of sickness absence from work in conjunction with the date of first *diagnosis* was recorded in 492 (12.4%) of the women and 170 (11.4%) of the men; the proportion of newly sick-listed women and men was 13.4% and 12.1%, respectively (Table 2). Overall, 1092 (31%) of the women and 346 (25%) of the men were on sick leave (new or ongoing) in conjunction with the date of diagnosis. Among the surgically treated CTS subjects, a new period of sickness absence from work related to the date of *surgery* was recorded in 1121 (53%) of the women and 408 (58%) of the men. The median duration of sickness absence from work was 35 days (IQR 27–45) for women and 41 days (IQR 28–50) for men. Overall, 1515 women (72%) and 523 men (74%) were on sick leave (new or ongoing) in conjunction with the date of surgery.

**Table 2:** Sickness absence from work in the carpal tunnel syndrome (CTS) cohort in conjunction with the dates of first diagnosis and surgery.

	All persons	Sickness absence from work^a^	Persons without full-time disability^b^	New sickness absence period^a^	Duration	
	N	N (%)	N	N (%)	Median (IQR)	Mean (SD)
**Women**						
Diagnosis	3966	1092 (31.4)	3675	492 (13.4)	40 (28–61)	73 (119)
Surgery	2111	1515 (71.8)	1950	1121 (57.5)	35 (27–45)	44 (44)
Any visit^c^	3966	NA	3675	1258 (34.2)	42 (28–70)	67 (91)
**Men**						
Diagnosis	1490	346 (25.4)	1406	170 (12.1)	44 (28–73)	98 (152)
Surgery	710	523 (73.7)	677	408 (60.3)	41 (28–50)	52 (65)
Any visit^c^	1490	NA	1406	448 (31.9)	46 (31–80)	80 (111)

^a^ Sickness absence from work from 15 days before to 7 days after the date of first diagnosis of CTS or date of carpal tunnel release surgery; a new sickness absence period is one that *started* within that time interval. The maximum possible sickness absence duration after first diagnosis for any individual in the study is 720 days.

^b^ Excluding individuals with 100% disability pension.

^c^ New periods of sickness absence from work during 24 months after the date of first diagnosis, occurring in conjunction with any doctor visit in which a primary diagnosis of CTS was registered.

## Discussion

Our population-based observational study shows that individuals with new physician-diagnosed CTS have significantly more days of all-cause sickness absence from work than individuals without CTS. The higher amount of sickness absence from work, shown as mean number of days of sickness absence per 30-day periods, existed 1 year before the date of first diagnosis and persisted up to 2 years after that date, peaking during the 2 months after the diagnosis. Although most sickness absence from work in the CTS cohort was associated with the carpal tunnel release surgery, even nonsurgically treated individuals had substantially more sickness absence days than the reference cohort without CTS.

In the CTS cohort, the higher amount of sickness absence from work beyond the few months immediately after diagnosis and surgery compared to the reference cohort may be related to associated comorbidities and/or a degree of disability that persists despite treatment. Women had a modestly higher level of baseline all-cause sickness absence from work than men and this continued beyond the immediate time after the diagnosis. However, no significant sex-differences were found in the number of days of sickness absence from work in conjunction with CTS diagnosis and surgery.

Following CTS surgery, most patients have work disability that varies in duration depending on the degree of postoperative morbidity [5]. The factors that may influence duration of postoperative work disability include possible wound complications, severity and duration of local pain and tenderness, type of work, patient expectations, comorbidities and even non-medical factors [9–14].

In our study the majority of the patients who did not undergo surgery within 24 months of CTS diagnosis did not have sickness absence periods exceeding 2 weeks. Data in Table 2 show that, of the nonsurgical subcohort, only 3.7% of the women and 2.9% of the men had a new sickness absence period in conjunction with any visit to a doctor in which a diagnosis of CTS was registered. In a large Spanish study of 13,000 persons sick-listed because of a musculoskeletal diagnosis excluding trauma and surgery during a 2-year period the proportion with CTS diagnosis was only 0.5% and median duration of sickness absence was 4 (IQR 1–10) weeks [15].

In the CTS cohort, the majority of sickness absence days belonged to the surgical subcohort and the increase in sickness absence started before surgery. A previous study found that patients who were absent from work because of CTS before surgery had significantly longer postoperative sickness absence than those with no sickness absence before surgery [5].

Our study has limitations. All sickness absence shorter than 15 days was not registered in the social security database and was thus not included, which may underestimate the proportion of sick-listed subjects in conjunction with diagnosis and surgery. The strengths of our study include the population-based matched-cohort design, inclusion of all health care, the physician-made diagnosis, and use of sickness absence data from the social insurance register rather than self-report. These factors enhance the generalizability of our findings.

## Conclusions

Persons aged 17 to 57 years with new physician-diagnosed carpal tunnel syndrome have significantly more sickness absence from work than age and sex-matched persons from the general population, from 1 year before to 2 years after the diagnosis. Differences in sickness absence between women and men were small. Information on work ability should be included in guidelines for treatment and rehabilitation in carpal tunnel syndrome.
